# Managed alcohol: one community’s innovative response to risk management during COVID-19

**DOI:** 10.1186/s12954-021-00574-5

**Published:** 2021-12-06

**Authors:** Heidi Brocious, Kathi Trawver, LaVerne Xilegg Demientieff

**Affiliations:** 1 University of Alaska Anchorage, Professional Studies Building, Suite 234, 3211 Providence Drive, Anchorage, AK 99508 USA; 2 University of Alaska Fairbanks, P.O. Box 756280, Fairbanks, AK 99775 USA

**Keywords:** Harm reduction, Managed alcohol program, Homelessness, COVID-19, Alcohol use disorder

## Abstract

**Background:**

Harm reduction programs often lack community-based support and can be controversial, despite data demonstrating effectiveness. This article describes one small Alaskan community’s development of a harm reduction managed alcohol program (MAP) in the context of a city-run quarantine site for individuals experiencing homelessness. The MAP was developed to support quarantining by COVID-19-exposed or COVID-positive individuals who also experienced chronic homelessness, a severe alcohol use disorder, and heightened health risks related to potentially unsupported alcohol withdrawal.

**Method:**

Five interviews with key informants involved in planning or implementation of the MAP were conducted using rapid qualitative analysis and narrative analysis techniques**.**

**Outcome:**

This study documents the planning and implementation of an innovative application of a managed alcohol harm reduction intervention in the context of the COVID-19 pandemic. In this instance, a MAP was used specifically to limit hospital admissions for alcohol withdrawal during a surge of cases in the community, as well as to mitigate spread of the virus. Key informants report no residents enrolled in the MAP program as a part of quarantine required hospitalization for withdrawal or for COVID symptoms, and no shelter resident left the quarantine site while still contagious with COVID-19. Additionally, the level of community support for the program was much higher than originally expected by organizers.

**Conclusions:**

This program highlighted an example of how a community recognized the complexity and potential risk to individuals experiencing structural vulnerability related to homelessness and a severe AUD, and the community at large, and was able to create an alternative path to minimize those risks using a harm reduction strategy.

## Introduction

In March 2020, the World Health Organization classified the emerging coronavirus disease (COVID-19) a global pandemic, prompting emergency implementation of numerous strategies intended to mitigate the spread of the virus [38, 43]. By September 30, 2020, the USA had confirmed over 950,000 COVID cases and nearly 200,000 deaths [54]. Emerging research quickly exposed that COVID-19 was impacting Americans inequitably, and was further deepening and worsening existing racial, social, socio-economic, geographic, and health disparities [1, 24, 28, 58].

Chief among the disadvantaged and marginalized groups impacted by COVID-19 have been Americans experiencing homelessness. Individuals who experience homelessness have contracted COVID-19 at disproportionately higher rates [27]. For example, an early study in Boston found that 10% of the city’s estimated homeless population had tested positive for COVID-19 with a 36% positivity rate within a 4-week period [2, 3]. Moreover, those who experienced homelessness have shown elevated risk of severe illness related to the disease due to advanced age, accelerated physical decline, lack of medical care, harsh living conditions, and other serious underlying medical conditions [7, 12, 18].

Residing in congregate shelter space or living without housing naturally limits control over one’s personal space, increasing potential exposure to the virus, and making isolation and quarantining highly challenging, if not impossible. Even when physical accommodations permit isolation, many people experiencing unsheltered homelessness and additional health and mental health needs require additional health and social supports to complete required quarantine requirements [7]. In response, communities across the globe developed congregate and non-congregate temporary accommodations to house those who have or were suspected to have COVID-19 but did not require hospitalization and were unable to self-isolate to quarantine and/or recover following a diagnosis [8, 55].

People who experience homelessness also have higher rates of trauma, physical and behavioral health challenges, and substance use disorders [6]. The rate of alcohol use among homeless individuals is up to 56% higher than the general population [17] with an estimated 38% considered to have a substance use disorder [49]. As a result, some alternative shelters quickly expanded implementation and use of harm reduction strategies.

### Managed alcohol programs

One type of emerging alcohol harm reduction strategy is the managed alcohol program (MAP). Originating in Toronto Canada in 1997 following three winter street deaths among that city’s homeless residents, MAPs have primarily operated within Canada, although some other European countries and Australia are beginning to develop programs [5, 16, 37, 42, 50]. Typically offered within housing programs or medical facilities, MAPs provide a low barrier alcohol harm reduction intervention targeting individuals with severe substance use disorders who experience homelessness and face barriers to accessing appropriate treatment [4, 32, 36, 41]. Typically, program participants are administered regulated doses of alcohol in hopes of stabilizing drinking patterns and reducing harmful drinking, often in conjunction with housing and other support services [10, 42, 51, 57]. MAPs are intended to address the myriad of vulnerabilities caused by “multiple intersecting harms,” “structural oppression,” and severe alcohol use among persons experiencing homelessness [, p. 58]. More specifically, MAPs target serious substance use-related health disparities including increased risk of disease, injuries, alcohol withdrawal, and death as well as facing barriers in securing housing and/or emergency shelter [26, 57]. MAPs are also implemented to preserve dignity and reduce other harms, including drinking non-beverage alcohol (e.g., hand sanitizer, mouthwash), binge drinking, drinking in unsafe settings, and experiencing alcohol withdrawal syndrome [4, 42]. Recently, researchers have begun to explore the need and feasibility of adding cannabis substitution in MAPs [39].

### MAP outcomes

Until recently, the available research literature was primarily limited to small scale evaluations focused on individual programs conducted by a group of Canadian researchers; however, the developing body of MAP evidence shows positive impacts of MAP related to a number of individual and community outcomes. For example, researchers have found MAPs stabilize and/or reduce alcohol use and reduce the consumption of more harmful non-beverage alcohol [29, 34, 45, 51, 53, 57] and drugs [14]. Researchers have also found overall improved health outcomes such as reductions in detox admissions, ambulance services, emergency room visits, and hospital admissions [34, 41, 45, 57]. Additionally, MAP participants experience fewer withdrawal-related seizures [34, 41, 45, 53, 57], as well as increased engagement with health and social support services [31], and improved medication adherence [15]. More broadly, MAP participants have reported feelings of safety, satisfaction with the program and living environment, a perceived improved quality of life, and positive impacts on their physical and mental health [36, 41, 52].

Related to community outcomes, researchers have identified MAPs as having a positive impact on housing retention [31, 41, 52] as well as reduced police [45, 57] and public emergency services contacts [16]. Finally, MAPs have shown significant community public safety and health cost savings [15, 21].

### Harm reduction and risk environmental framework

At their core, MAPs are grounded in harm reduction principles and a Risk Environmental Framework (REF). A discussion of both, harm reduction and REF, and how they are connected, is important to understand the complex nuances of MAPs. They also provide a lens upon which to understand the managed alcohol program intervention discussed in this study and will be utilized to frame the discussion of outcomes at the end of this article.

Harm reduction is a public health-based approach to reduce adverse health, social, and economic consequences of substance use and other risky behaviors as an alternative to requiring complete extinguishment of the behavior [22]. Thus, using a harm reduction approach related to substance use, the treatment is not contingent on complete abstinence and its target is not the use of drugs and/or alcohol per se, but rather the negative impacts of use. According to Hawk et al. [22], the philosophy and principles of harm reduction include humanism, pragmatism, individualism, incrementalism, autonomy, and accentuality without termination. Further, this framework values the lived experience voice in creating programs and recognizes that social injustices and inequalities (e.g., racism, poverty, classism, discrimination) impact substance use vulnerabilities and capacity to mitigate its harms [33]. Reported strategies related to substance use include increased access to medication assisted treatment (i.e., Methadone, Naloxone), telehealth, take home and prescription delivery, supervised consumption services, and needle syringe programs (e.g., [23, 27, 30, 35]. Well before the emergence of COVID-19, a strong body of literature affirmed that harm reduction services were effective in reducing the spread of diseases without increasing the use of alcohol or drugs (e.g., [9, 19, 44, 46].

Risk Environmental Framework expands on the theory and principles of harm reduction theory by generating an understanding of the complexity of harm reduction efforts through exploration of risk environments. In other words, this framework emphasizes that risk is developed across all levels of person-in-environment interactions (micro to macro) within the context in which people live [47, 48]. Examples of risk environments (REs) at the microlevel incorporate the context the individual lives in to include, living in poverty, being unhoused, lack of a support system, peer group and social influence, experiencing racism and/or other isms, having a substance use disorder and/or a co-occurring mental health disorder, health issues, impact of societal norms and values, and other vulnerabilities. Examples of REs at a macrolevel include multiple types of inequity and oppression in policy and practice within systems of care to include health, legal, economic, and social service systems to name a few, as well as historical and generational trauma. Substance use harms are shaped by risk environments [48]. A REF additionally requires an acknowledgement that systemic and structural inequities are responsible for both creating and reducing risk and harm with vulnerable populations [40, 47].

As Glass and McAtee [20] describe, “Health behaviors occur in patterns because they are shaped by social factors residing at levels of organization above the individual, in conjunction with the consequences of biological systems within the body” (pp. 1655–1656). An exploration of the complex dynamics and patterns of this person in environment interaction and its impact on risk can help shape understanding of how interventions and programs, like what is discussed in this study, work to decrease risk and harm in ways that target both individuals and the systems in which they live.

### Purpose and approach

Despite strong empirical support and widespread use of harm reduction interventions across Canada, Australia, and Europe, the USA has been slower to adopt MAPs (e.g., [9, 19, 41, 44, 46]. While prior to COVID-19, no known MAPs were operating within the USA, providers in San Francisco, California recently reported implementing use of the model with the city’s homeless services [31]. Also in response to COVID-19, the city of Juneau, Alaska, implemented a MAP program with a narrow focus, designed to reduce the spread of the virus by individuals experiencing homelessness and serious alcohol use disorders.

## Methods

### Data collection and analysis

Because the goal of this study was to describe the newly developed program and the perceived impacts quickly so that other communities facing similar pandemic concerns could benefit, study authors employed and adapted rapid qualitative analysis (RQA) techniques to collect and analyze the data collected in this study [59]. One key goal of RQA is to reduce the time spent collecting and analyzing data when there is a pressing need to share information quickly. RQA techniques can include analysis without creating transcripts, “mind-mapping” or other tools to reduce the time from data analysis to publication [59]. In this study, RQA techniques were used in combination with narrative analysis techniques of organizing data for ‘restorying’ where different narratives are brought together by the researchers to place their shared information in chronological order, following traditional aspects of storytelling [11].

Specifically, in this project, stakeholder interviews were recorded and transcribed. Two authors then listened to the recordings and reviewed the generated transcripts, developing both a story and timeline that integrated different perspectives on the creation, implementation, and perceived impact of the shared story. Further, to gather community reactions to the MAP program, authors searched for relevant Facebook posts and comments on both the official City page, as well as a popular community Facebook page used by more than 18,000 community residents. All posts and subsequent community member comments made during the time in which the MAP program was operational were scanned for relevant content.

### Study participants

Five participants who were involved in either the planning or implementation of the project were interviewed for this project in December of 2020. Interviews occurred over a HIPAA compliant Zoom link and lasted between 30 and 60 min, depending on how much participants wanted to share. Two were administrators or managers of the local hospital, one was a member of the local city government, and two were individuals who worked as fulltime direct care staff at the alternative shelter site. Three of those interviewed were directly involved in administering alcohol to individuals. Two of the interview participants were licensed health care providers. While the perspective of participants (those who received administered alcohol) would have been an illuminating and useful addition to this study, the decision was made not to interview program participants due to the length of time it would have taken to establish a participant data sharing agreement with the City, and the acknowledged difficulty in locating these individuals several months after the conclusion of the service. Institutional review board approval was obtained from the University of Alaska Anchorage.

## Results

### MAP development

Located in the southeast panhandle of the state, the small city of Juneau serves as the Alaska state capital. *Dzántik'i Héeni* (Base of the Flounder's River in Tlingit), or Juneau, is home to just under 32,000 people [56] and is only accessible by boat or air, having no roads that connect the city to the rest of the State. During the January 2020 homeless point-in-time count, a total of 244 adults were identified as homeless including 46 unsheltered, 82 living in transitional housing, and 116 staying in emergency shelter [25]. In March of 2020, like other communities around the world, community leaders assembled an emergency operations team to respond to the emergent COVID-19 outbreak including members of local government, administrators from the one local hospital, the one State public health office, and local fire, ambulance, homelessness service providers, and facilities/building maintenance. One task of this group included how to address quarantine and isolation needs if the virus spread among community members experiencing homelessness.

Members of the team responsible for developing a plan for individuals who were homeless formed a special task force. The task force was keenly aware from the onset that issues related to substance use would be a primary challenge among the population. Even in the earliest meetings, general discussions began about how they might manage substance dependence among those without housing in the context of city supported alternate care sites.

Fortunately for the community, case counts of COVID-19 were comparatively lower than in many other areas of the world and remained relatively low throughout the spring and early summer of 2020. The task force charged with responding to COVID-19 among those experiencing homelessness initially secured a school gym to temporarily serve the small numbers of individuals who needed to quarantine while awaiting test results. Fortunately, none of the individuals housed in quarantine at the gym tested positive or required isolation, and no issues around substance use or withdrawal presented themselves during these early days. Once this small number of initial suspected cases was managed, the city closed the temporary gym location and began to look at other more accessible and better-equipped locations.

Despite the absence of early cases, the task force continued to anticipate potential challenges. As the local rates of COVID-19 began to grow over the late summer of 2020, the task force secured a larger, more centralized homeless quarantine shelter at the city-owned convention center. Since opening a large quarantine center required extensive setup and staffing, the first few cases of COVID-19 among those experiencing homelessness were managed through a contract with a local hotel that could provide a handful of rooms for individuals to isolate. The city then worked directly with the hotel to coordinate safety protocols and food delivery. This temporary solution was met with a great deal of tension, as hotel staff had difficulty monitoring isolation, and conflict often arose when the quarantined residents attempted to share their temporary housing with others in the community who had nowhere to stay. One participant noted:

The management at the [hotel] were very concerned about the evolving situation and their staff are not medical professionals. They don’t know how to manage it. They just see people coming and going. They didn’t know who was positive, who was not, and they really kind of hit a wall and their ability to manage situation.

It was during this time that an individual who was quarantining in one of the secured hotel rooms was admitted to the hospital with severe alcohol withdrawal symptoms. He proceeded to have a complex, medically managed withdrawal, complicated by his COVID-19 diagnosis, and required several weeks of hospitalization. With no local, medically managed withdrawal services in the community outside of this small regional hospital, the case highlighted the need to have an alternative system in place to avoid filling hospital beds with patients who were withdrawing from alcohol. At this point, the task force began to discuss in earnest how they would manage the risk of alcohol withdrawal and began looking at the academic literature on managed alcohol programs already in place in other communities. Swiftly and collaboratively, hospital administrators, city officials, emergency room providers, and community emergency service workers determined that a plan that provided alcohol withdrawal prevention or management through the supervised administration of alcohol was the best course of action.

### MAP implementation

While the one withdrawal-related hospitalization highlighted the need to act swiftly, the task force had already laid the groundwork for implementing a harm reduction strategy. Early on, they worked through concerns from members who were less familiar with alcohol dependence, educating them about why alcohol withdrawal is not just uncomfortable, but potentially life-threatening and often requires hospitalization. Members of the task force who were familiar with harm reduction also held early discussions to address potential legal concerns around the liability of a government organization both purchasing and providing alcohol, and protocol concerns about how to prescribe and administer alcohol. One stakeholder noted:

We wanted to bring them articles about this being done in other places. People were worried about what would happen if more harm came to people after we administered them alcohol – like what would happen if the city gave someone alcohol and then they had a negative outcome?

They went on to explain that existing peer reviewed literature on the approach was used with skeptical city officials to demonstrate that MAPs were in fact in practice in other communities.

While planning for a MAP approach was still discussed, Juneau experienced its first significant COVID-19 outbreak among community members experiencing homelessness. At the same time, it became clear that the hotel contract was neither working effectively, nor were there enough rooms to meet the growing need. A decision was made to quickly move the small number of residents temporarily housed at the isolation hotel to the larger community convention center. Given the speed with which the new shelter site needed to be set up, the City collaborated with the local hospital to temporarily staff the site. Registered Nurses acted as “site supervisors” and behavioral health techs and other medical paraprofessionals, along with members of the city task force, staffed initial shifts at the facility.

At this point, the task force was actively working with medical providers and had discussed a general protocol for MAP implementation. However, the mechanisms and details of this protocol were not yet developed, and this lack of detail raised several questions, such as Who would buy the alcohol? Where and how would the alcohol be stored? What tool would be used to assess need? How would this be documented?

While task force members were drafting protocols, the need to immediately implement the program presented itself. One city official and task force member reported that, “within six hours [of opening the isolation quarantine site] there was somebody actively detoxing, and the skilled nursing didn't really want to watch that happen.” After a brief consultation with the medically licensed members of the team, one task force member went to the local liquor store, purchased some alcohol using his personal money, and administered a measured shot of alcohol to the COVID-19 positive shelter resident entering active withdrawal; the individual quickly improved. This marked the beginning of administering small amounts of alcohol to COVID-19 positive individuals to both prevent withdrawal and support residents’ ability to quarantine by eliminating any need to leave the center to obtain alcohol. A study participant noted:

We couldn't run the risk of people having an adverse event because they didn't have alcohol or taking upon themselves to voluntarily leave and go out into the community with a communicable virus. Okay, so we were trying to ensure that they would stay there and that their needs were met, you know.

Study participants felt that by providing medically recommended amounts of alcohol to alternative shelter residents this intervention reduced both the risk of withdrawal and the risk of increased community transmission by COVID-19 positive patients.

As the number of individuals at risk of severe alcohol withdrawal and needing quarantine shelter increased, the task force set about finalizing MAP administration protocols. Up to this point, the City and the Hospital had already consulted with their legal departments about risk management concerns. Interviewed stakeholders reported that legal counsel acknowledged the unique circumstances COVID-19 presented, as well understanding that a MAP approach provided for less risk than did the alternatives of hospitalization or failure to isolate while positive for COVID-19. An emergency room doctor who was consulting with the task force issued a standing set of orders, a general “prescription” for up to four two-ounce doses of whiskey or vodka to be administered every 24 h, three with meals, and the remaining dose available as needed.

Participants reported that the task force adopted a set of screening questions to determine MAP eligibility: (1) Do you drink alcohol?; (2) Do you drink more in the morning to stave off withdrawals?; (3) How much time passes between drinks before you begin to go into withdrawal?; (4) Have you ever had a seizure? If yes, was it while you were withdrawing from alcohol?; and (5) What are your goals related to your alcohol use? Do you want to cut back? Individuals answering “yes” to both question “1” and any of questions “2” through “4” were determined eligible for the program. During assessment, if an individual was believed to already have alcohol in their system, either because they had just arrived and endorsed use or demonstrated significant slurred speech, unstable balance, erratic behaviors, and a strong smell of alcohol, the protocol allowed the team to delay administering doses of alcohol. If site staff or the site manager were ever in question about the appropriateness of administering a dose of alcohol, they were able to call on city Emergency Medical Technicians (EMTs) to come and assess the patient for levels of intoxication or withdrawal. One participant commented on the decision to co-locate the local EMT COVID-19 response team in the ballroom in the same building as the temporary quarantine site:

[the EMTs] are now on one of the conference rooms and so that added, you know, another 24 seven medical presence and it's like a home base for the mobile integrated health and care so they'll stop by as needed on routine checks…it has been great to be able to communicate between, you know, [quarantine shelter staff] and mobile integrated health to say hey you know something's going on. Maybe you can go and check on this person. And it's been great, you know, they just stopped by and it's been really beneficial for the site assistance and the site managers to know they're there in the neighborhood too.

Hospital officials quickly created a data collection system that included the ability to document a person’s withdrawal risk at admission, MAP eligibility, alcohol administration, resident symptoms related to both COVID and withdrawal, and instances of community EMT calls to check on or monitor patient symptoms.

### Successes and challenges

Despite initial task force concerns, there were no negative reactions from the larger community about the MAP. The task force proactively informed the EOC leadership and city officials about the program and armed them with talking points they could use if they received constituent phone calls about the program. The city authored a press release that was posted to their Facebook page. Somewhat surprisingly, there was very little response, garnering just 11 comments, all of which were positive and complementary of the city’s efforts. One commenter even stated, “wise use of harm reduction strategies” as her reaction to the program overview.

Several factors seemed to have contributed to community acceptance and quick implementation of the MAP harm reduction approach. In this case, necessity may have become the mother of implementation. First and foremost, the gravity of the COVID-19 pandemic had already highlighted the need for a “whatever it takes” mindset, perhaps allowing for initiatives and interventions that would previously have been viewed as unacceptable. Within the context of transitioning public schools to online learning, rapidly shifting business strategies, and most community members working from home, the community’s expectation was already in place to understand and accept that what was once taken for granted would need to be addressed differently. Second, despite having no prior plans to implement a MAP, interviewed task force members reported that because they talked “early and often” about the challenges substance use, and in particular alcohol use, would have on residents who were chronically homeless, it may have served to prime all members to act quickly when action was required, enabling consideration of a MAP as a part of a viable and rapid response. Finally, with a community limited to 57 regional hospital beds, with the next closest option requiring a medical evacuation and a two-hour flight, one of the most pressing goals for the community was to keep hospital beds as free as possible to be ready to meet the need should there be a surge in COVID-19-related hospitalizations.

Despite this success, implementation was not free of bureaucratic challenges, the first of which being how to purchase alcohol. Corona Virus Aid, Relief, and Economic Security Act (CARES) funding prevented the purchase of alcohol, and long-standing city policy and procedures also restricted the purchase. After consultation with city finance, it was decided that the best route was to use the city petty cash system and city general funds, which allowed for internal approval and did not require the use of city purchase orders, checks, or purchase cards. The task force received administrative level support for a short-term approval to purchase alcohol, and one task force member took the lead on purchasing and seeking reimbursement through the EOC approved process.

### Resident reactions

It was not surprising that a broad concern among stakeholders was how residents of the quarantine shelter facility would react to having alcohol available. Study participants wondered: Would there be conflict between residents on the alcohol protocol and those who weren’t? Would it be challenging to set limits on the amount of alcohol that could be administered? Would there be pushback they would have to manage regarding access to other legal drugs such as tobacco and cannabis? How would they know if residents were only taking the staff provided alcohol and not consuming their personal alcohol? Several participants described a vague feeling of holding their breath waiting for something to disrupt the process. One task force member noted that there was an initial period where alternative shelter staff felt some of their concerns were materializing:

By the next morning word on the street was that there was alcohol being provided at Centennial Hall, and so people would just be walking by the parking lot and coming up to the outdoor area and saying, hey, can I have some and so we had to be like, no, we're, we're working on the protocols to make sure that it's for this program and prescribed by a health care physician.

Despite initial participant concerns that people might seek to abuse the system, this did not occur. Another study participant, one who provided direct services noted:

I'll just tell you, overall, like I remember it being this big deal like, oh you know even when I would tell people that I know like, well, we're giving people shots of alcohol at this center people would say “wait you're doing what?” you know? But my overall impression was we never had any issues, really. I mean, there was nobody that I saw abusing the protocol and it kept them happy. And it kept them there. We never had anybody go into withdrawals. It seemed to work really well.

Shelter staff noted that some residents asked why the city did not also provide cannabis. However, after a few days even these gentle pushbacks ceased, and the general, and the general response to the MAP, both from those receiving the protocol and those who were not, was gratitude for the option. Once alternative shelter residents understood there was a screening process and medical reasoning behind administering the protocol to some residents, requests to be issued alcohol by non-protocol recipients reportedly diminished. Staff had to remain alert to new shelter residents bringing in alcohol with them and adjusted alcohol doses accordingly. Shelter staff further reported that they had strong support from non-drinking residents who were extremely supportive of the provision of alcohol for their friends who needed it. Once site supervisor stated:

I will say one thing, okay, that residents were hugely supportive of those people who needed to be on the protocol being on the protocol. So, there was a little situation apparently overnight. There was a sick gentleman...… and he was extremely symptomatic when I saw him first thing in the morning and his peers who were there with them were so upset that he had not been given alcohol at like a four o'clock in the morning.

Another factor that likely mitigated negative responses from residents was the overall philosophy and approach by the staff. Under the leadership of the task force chair, and because all participation was voluntary and COVID-19 positive residents could leave at any time, the mission of the quarantine facility was to make residents as comfortable as possible so they would choose to stay, keeping themselves isolated and the community safer. The shelter staff developed an outdoor smoking area and provided cigarettes to residents. The outdoor area was setup with double fencing so that residents could be outside, could even see friends or family, but the second set of fencing was set more than six feet out so that quarantine residents and passersby remained physically distanced. Residents that had special food requests were also accommodated, and books, games, and television were all provided to encourage COVID-19 positive alternative shelter residents to remain in isolation for the 10–14-day period. Task force members described how this responsiveness served to build positive relationships with alternative shelter residents and that some were making independent decisions to scale back even the small amount of alcohol they received. Indeed, one site staff member stated:

I noticed that there were people who, from the time they started to when they left, that they kept on their four shots, but there were some that weren’t by the time they left, they weren’t doing as much. We talked to quite a few of them about going into treatment.

Another study participant noted that the client-centered, needs focused way the entire program delivered was different from many of the more restrictive or even punitive systems alternative shelter residents engage with on a regular basis. She noted:

…There's already a sense of defensiveness and push away... but when they can be humanized again --- I think that there's going to be all sorts of benefits --- for everyone. When you can relate to people and connect with people, it is a way of building connection and relationship and trust that and also that people matter, having people have a sense that they matter. That's only going to help them, you know, with the choices that they're making in their lives when people feel better about themselves or feel that connection --- you have so much more potential.

From this participant’s perspective, the program model allowed alternative shelter residents to experience a staff committed to meeting resident needs wherever possible, and believed staff held a genuine concern for the health and well-being of residents. Additional study participants noted they felt this foundation of meeting resident needs could be linked to high compliance, low conflict, and possibly even created the space for some residents to choose to cut back on their alcohol consumption.

### Impacts

Through frequent COVID-19 testing and providing a quarantine site for those who needed to isolate, the virus was successfully managed as envisioned. In total, the Centennial Hall quarantine shelter operated for eight weeks serving 37 individuals, nine of which were provided MAP services. Of those housed, none required hospitalization for either alcohol withdrawal or COVID-19 symptoms. Additionally, no COVID-19 positive shelter residents decided to leave the facility prior to the end of their quarantine period, experienced seizures, or any significant withdrawal symptoms. Local hospital beds were protected during a significant outbreak among individuals experiencing homelessness. During the alternate shelter operation, extensive testing was conducted each Friday to ensure that members of the homeless community as well as the 35–40 staff that were employed at the shelter remained COVID-19 negative. During the eight-week period, no staff tested positive and no new cases were identified among the unhoused. Finally, after approximately two weeks without any new cases among those experiencing homelessness, the site was able to close (but remains ready to be quickly re-opened if needed).

All of these successful outcomes were possible due to a complex systems approach on many levels. Rhodes [48] discusses risk as being socially situated and that a variety of factors on multiple levels are interacting to increase or decrease risk. In this study, there were several individuals from a variety of systems that worked together to mitigate and decrease risk. A discussion on physical outcomes is shared above, however, there were additional benefits that emerged in other areas. Economically, this intervention had the potential to save on extensive medical costs had there been a surge in COVID19 related hospitalizations, not to mention social-emotional costs to families and community members. The social-relational impacts highlight important relationships and connections made between service providers and between service providers, the unhoused individuals, and local community members. These relationships created valuable awareness, increased compassion, and decreased stigma related to those who are unhoused and experience high needs. Finally, politically, there were individuals who navigated this challenge in a way that decreased tension and found solutions around policies where before there were long-standing barriers. Although the COVID-19 pandemic created and highlighted inequities in health care and access to services for many vulnerable populations, community leaders took all these moving parts into account and found a real and innovative solution.

## Discussion

The purpose of this inquiry was to describe the implementation of a short-term emergency COVID-19 response that incorporated the principles of harm reduction. The goal of the temporary shelter embedded MAP was to prevent avoidable hospitalizations while protecting the health environments of the general community and shelter residents alike. Interestingly, a few other benefits also emerged. This program is an example of a narrower application of MAP principles as MAPs typically provide long-term alcohol management, supportive housing services, and are not connected to emergency COVID-19 quarantine efforts [10, 42].

The REF is discussed as a human rights issue, one of the goals to make visible to the broader society the physical, social, political, and economic dimensions of risk, rather than focus solely on personal responsibility for change in risk conditions and health outcomes [13]. Utilizing the REF to examine the impacts of the MAP, we are able to view how multiple parts of the environment, individuals, organizations, and the community as a whole, to include attitudes, practices, and policies, interacted to decrease health risk within a vulnerable population in a small rural city in Alaska during a pandemic.

Several key lessons learned during this project highlight the REF model of understanding risk, ones that may be useful to other communities considering the implementation of both emergency and longer-term harm reduction strategies. First, collaborative relationships among task force members were key, as was talking early and often about the potential problem. By beginning to discuss this early on in the process and educating members about the risk and philosophy of harm reduction, the team was conceptually ready to implement it very quickly when it became necessary, as well as kept creating new and better strategies when prior attempts were lacking. This highlights the REF concept how context can shape risk, and how the intentional engagement and education of mid-level policy makers can change the risk context [48].

One surprising lesson from this program implementation was that, when need demands it, a bureaucracy can successfully mitigate stigma, work around rigid rules like those prohibiting the purchase of alcohol with public dollars, and innovate to significantly protect individuals and public health. This exemplifies the REF principles of both economic and policy risk at the macrolevel, where newfound flexibility in both implementation policies and funding access can be attributed to the dynamic policy changes that came with COVID-19. Indeed, one general lesson from this global pandemic may be that we as collectives and as institutions are more flexible than we previously thought. Relatedly, because of these proactive and preventative actions there were no major community tragedies, such as death from withdrawal, shortage of hospital beds, and no surge in COVID-19 among the unhoused or healthcare staff, which undoubtedly saved lives and saved money.

The REF is also useful in understanding why there was little to no negative community reaction to the MAP program. As noted, any expected community backlash was muted or non-existent. This may be due to an enhanced sense of connection that community members made between their own health and well-being and those residents experiencing homelessness and alcohol dependence, making it much easier to see how individual and community health are inextricably linked. In real time, community members who had previously not viewed their physical environment as interdependent with people in their community experiencing homelessness were easily about to see the mutual benefits of a harm reduction approach.

In this case, the harm reduction approach did not produce the negative consequences any detractors may have feared (e.g., encouraging alcohol use). Instead, the use of a simple, logical protocol that was accepted by a high-risk audience who responded positively to this approach which recognized and honored their needs, their free will, and humanized their experience.

Direct care staff reported that individuals staying in the shelter felt cared about and in the same predicament as everyone else dealing with the challenges of the pandemic, creating a feeling of commonality which seemed to promote mutual respect between both staff and residents, and between residents, who actively looked out for each other. The healthcare providers also recognizing this as a challenging time for everyone were more compassionate and less judgmental in their interactions adding to feelings of solidarity and support, which added to the program’s success.

### Limitations

By design, this program description was not intended as a comprehensive inquiry. The implications are limited by the short time frame of the project as well as the inability to talk directly with participants of the program to garner their perspectives. However, this program narrative provides a discussion launching point, particularly in the context of learning how harm reduction strategies can garner support when traditionally much of the USA has resisted this type of approach. Additionally, as stated previously, it is important to note that this study reflects a narrow use of the MAP model (one focused on preventing hospitalizations and promoting quarantine among COVID-19 positive individuals, so findings may not be transferable to more comprehensive MAP programs).

### Implications and recommendations

Planning and relationship building played primary roles in the successful and speedy launch of the described MAP. Additionally, the pandemic had primed many of the more rigid institutions to become more flexible and creative, setting the stage for introducing this innovative model**.** One of the most interesting aspects of this project was the lack of resistance it faced, presumably because community officials and residents could easily connect their own health and well-being to successfully quarantining COVID-19 positive individuals. Further, shared anxiety about limited hospital beds made nearly everyone interested in successfully diverting admissions. Future research exploring the potential connection between a shared sense of benefit as a strategy for garnering community support for controversial harm reduction programs may be useful to forward these strategies within the USA**.**

## Data Availability

Due to confidentiality concerns and the inability to de-identify the qualitative data the original interview transcripts are not publicly available.

## Abbreviations

MAP: Managed alcohol program
COVID-19: Coronavirus disease 2019
REF: Risk Environment Framework
EOC: Emergency Operations Command
EMT: Emergency Medical Technician
CARES: Corona Virus Aid, Relief, and Economic Security Act of 2020
